# P-1589. Home Infusion Short Course Remdesivir for Solid Organ Transplant Patients with COVID-19

**DOI:** 10.1093/ofid/ofaf695.1768

**Published:** 2026-01-11

**Authors:** Grace DeMarco, Rebecca Nirmal Kumar, Danial Mahmood, Alok Nimgaonkar, Joseph G Timpone, Rohit Satoskar, Princy N Kumar

**Affiliations:** Medstar Georgetown University Hospital, Washington, District of Columbia; Georgetown University School of Medicine, Washington , DC; MedStar Georgetown University Hospital, Washington, District of Columbia; Medstar Georgetown University Hospital, Washington, District of Columbia; Medstar Georgetown University Hospital, Washington, District of Columbia; MedStar Georgetown University Hospital, Washington, District of Columbia; Georgetown University Medical Center, Washington, District of Columbia

## Abstract

**Background:**

Solid organ transplant recipients (SOTR) are at high risk for severe COVID-19 infections due to immunosuppression and variable response to immunization. The recommended oral antiviral, paxlovid has significant drug-drug interactions with medications that prevent graft rejection. As an alternative, three-day intravenous (IV) remdesivir is efficacious in the outpatient setting. MedStar Georgetown Infectious Disease (ID) Division and Transplant Institute collaborated with an infusion company to administer remdesivir at home for SOTR to reduce hospitalization while avoiding severe COVID infection, drug interactions, and exposure of vulnerable patients in infusion centers. Here, we describe the feasibility, outcomes, and cost savings of short course remdesivir home infusions for SOTR with COVID-19.

**Figure:**
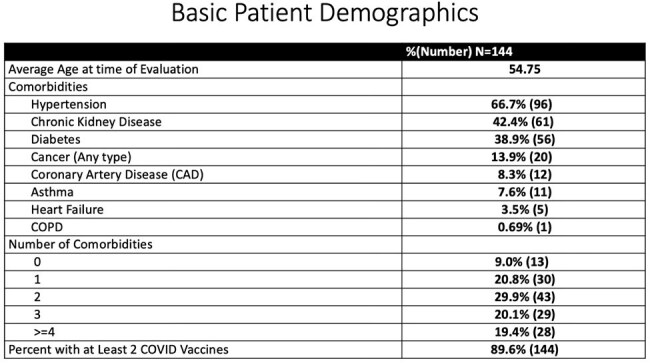

**Figure:**
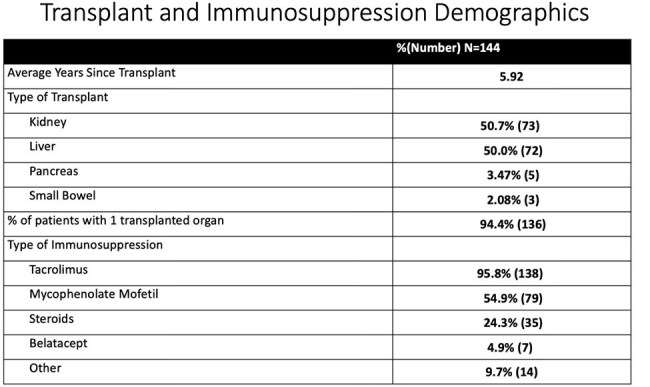

**Methods:**

We conducted a single center retrospective chart review study at MedStar Georgetown University Hospital of SOTR diagnosed with COVID-19 and referred to receive home IV remdesivir December 1, 2022 to May 6, 2024. Patients were evaluated by ID physicians, then started on a three-day course of remdesivir as an outpatient. Analyses included descriptive statistics of study participants’ demographics and outcomes. Costs of average hospital length of stay in Washington DC were compared to those of home infusion administration.

**Figure:**
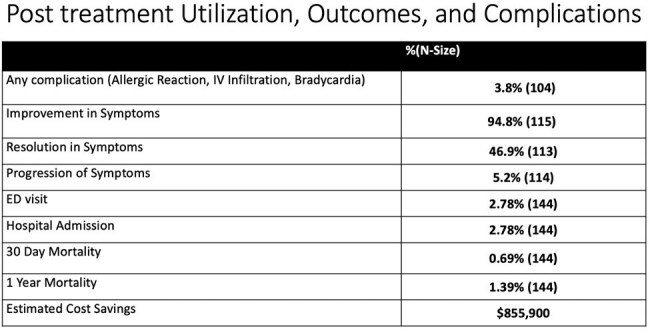

**Results:**

The study included 144 SOT patients: 50% liver, 50.7% kidney, 3.5% pancreas, and 2.5% small bowel. The average age was 55 years old, with an average duration of COVID symptoms of 2.5 days prior to presentation. 2.78% of patients were hospitalized within 30 days of evaluation, and 0.69% patient died. The average cost of administration for 3 days was $4279, comparable to one day of hospitalization in DC ($4068), saving an estimated $855,900.

**Conclusion:**

Patients receiving home infusion remdesivir had low hospitalization and mortality rates, suggesting reduced risk of disease progression. This program likely saved $855,900 by avoiding hospitalization. Limitations include an inability to compare actual costs and outcomes to a relevant inpatient population. Home infusion of remdesivir is a safe delivery method and may reduce overall cost of healthcare.

**Disclosures:**

Rebecca Nirmal Kumar, MD, AstraZeneca: Advisor/Consultant|AstraZeneca: Grant/Research Support|Pfizer: Grant/Research Support Rohit Satoskar, MD, Madrigal: Honoraria

